# Transcriptional Profiling of the Probiotic *Escherichia coli* Nissle 1917 Strain under Simulated Microgravity

**DOI:** 10.3390/ijms21082666

**Published:** 2020-04-11

**Authors:** Jaewoo Yim, Sung Won Cho, Beomhee Kim, Sungwoo Park, Yong Hee Han, Sang Woo Seo

**Affiliations:** 1 School of Chemical and Biological Engineering, Seoul National University, 1 Gwanak-ro, Gwanak-Gu, Seoul 08826, Korea; jaewoo95@snu.ac.kr (J.Y.); swon0912@snu.ac.kr (S.W.C.); bluesia@snu.ac.kr (B.K.); sw.park@snu.ac.kr (S.P.); 2 Interdisciplinary Program in Bioengineering, Seoul National University, 1 Gwanak-ro, Gwanak-Gu, Seoul 08826, Korea; wyongh@snu.ac.kr; 3 Institute of Chemical Processes, Seoul National University, 1 Gwanak-ro, Gwanak-Gu, Seoul 08826, Korea; 4 Institute of Engineering Research, Seoul National University, 1 Gwanak-ro, Gwanak-Gu, Seoul 08826, Korea

**Keywords:** microgravity, *E. coli* Nissle 1917, probiotics, RNA-sequencing

## Abstract

Long-term space missions affect the gut microbiome of astronauts, especially the viability of some pathogens. Probiotics may be an effective solution for the management of gut microbiomes, but there is a lack of studies regarding the physiology of probiotics in microgravity. Here, we investigated the effects of microgravity on the probiotic *Escherichia coli* Nissle 1917 (EcN) by comparing transcriptomic data during exponential and stationary growth phases under simulated microgravity and normal gravity. Microgravity conditions affected several physiological features of EcN, including its growth profile, biofilm formation, stress responses, metal ion transport/utilization, and response to carbon starvation. We found that some changes, such as decreased adhesion ability and acid resistance, may be disadvantageous to EcN relative to gut pathogens under microgravity, indicating the need to develop probiotics optimized for space flight.

## 1. Introduction

The recent surge of long-term space exploration necessitates an increased attention on the health risks associated with space travel. Studies have shown that astronauts who stay in space for extended time undergo changes in their microbiome environment. A study conducted by Garrett-Bakelman et al., at the National Aeronautics and Space Administration (NASA), has shown that an astronaut who underwent a year-long space mission experienced a change in his gut microbiome composition, compared to that of his earthbound twin sibling [1]. Furthermore, astronauts who have spent six to twelve months at the International Space Station (ISS) have also undergone changes in their gut microbiomes [2]. These changes include the increase of *Parasutterella*, which is associated with chronic intestinal inflammation with inflammatory bowel disease (IBD) and the reduction of intestinal *Fusicatenibacter*, *Pseudobutyrivibrio*, and *Akkermansia*, which have anti-inflammatory properties [2]. These results demonstrate that some factors during spaceflight can be critical to the gut microbiome of astronauts. These factors may affect the growth of both beneficial and detrimental microbial strains in the gut and thereby increase the possibility of the development of IBD.

Multiple studies have indicated that microgravity (MG) conditions induce several physiological changes. Studies conducted at the ISS primarily targeted pathogens such as *Salmonella enterica* serovar Typhimurium χ3339*, Bacillus subtilis* strain 168, and *Pseudomonas aeruginosa* PAO1, and have shown that virulence responses increase with enhanced stress resistance [3,4,5,6]. Unfortunately, due to the challenges of sending bacterial samples to the ISS, the majority of studies have been done in laboratories on Earth using a rotary cell culture system (RCCS) and simulated MG conditions. The application of the RCCS system has expanded the scope of target strains considered to include *Escherichia coli* K-12 MG1655 and others. The results obtained using simulated MG are similar to those from the ISS. In both cases, MG alters biofilm formation, starvation metabolism, metal ion uptake, and multiple stress responses, including acidic, heat, osmotic, and oxidative conditions of the microorganisms [7,8,9,10,11,12]. Although previous studies have focused on pathogens and laboratory strains [3,6,13], it is also important to investigate the effect of MG on beneficial strains, such as probiotics in the gut. One such strain is *E*. *coli* Nissle 1917 (EcN), which was isolated from feces of a German soldier and is a representative probiotic that has been reported to improve host immunity [14,15,16,17]. Its probiotic effects have been established and has been commercialized. For instance, it is the probiotic used in Mutaflor, which is medically effective in treating chronic constipation and Crohn’s disease, a type of autoimmune disease [18].

In this study, we performed comparative transcriptomic profiling of EcN to determine the effect of MG on cell growth and metabolism. We compared genome-scale data from strand-specific massive parallel complementary DNA (cDNA) sequencing (RNA-seq) during exponential and stationary growth phases under simulated MG and normal gravity (NG) conditions. This study should serve as a starting point for understanding of the effects of MG on the cell metabolism of probiotics and to provide insight into the engineering principles of probiotics, which will lead to an improvement in the health of astronauts during spaceflight.

## 2. Results and Discussion

### 2.1. Growth and Transcriptomic Profiling of EcN under Simulated MG and NG

**Figure 1:**
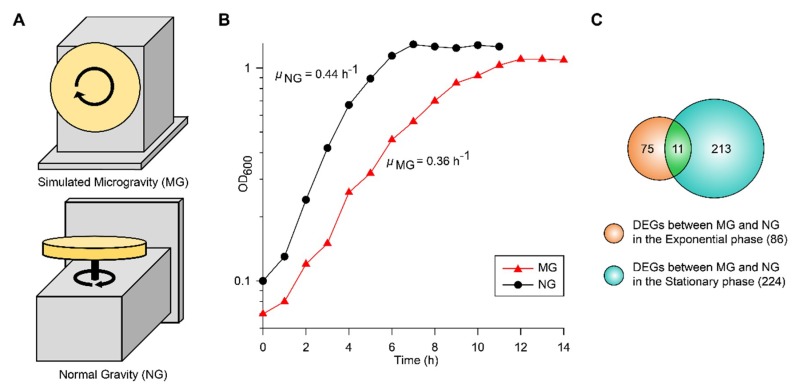
(**A**) The operating orientation of the rotary cell culture system (RCCS) under microgravity (MG) and normal gravity (NG) conditions is shown. (**B**) The growth profiles of EcN at 37 °C in a clinostat under MG and NG conditions are shown. (**C**) Identified differentially expressed genes (DEGs) during the exponential and stationary growth phases are shown in the Venn diagram.

To culture EcN under simulated MG and NG conditions, we used a clinostat with different directions of rotation (Figure 1A). MG conditions were simulated by rotating the system around the horizontal axis and manipulating the rotation speed of the vessel to reduce shear stress [19]. NG conditions were maintain by rotating the system around the vertical axis [9]. When EcN growth in glucose M9 minimal media was measured under MG (μ_MG_) and NG (μ_NG_) conditions, the maximum specific growth rates were 0.36 and 0.44 h^−1^, respectively (Figure 1B). In contrast to other studies that used *E*. *coli* K-12 MG1655 [7,9], the growth of EcN was slower under MG than NG. The growth difference under MG compared to that under NG may have been due to the sum effect of several factors involved with cell fitness.

**Figure 2:**
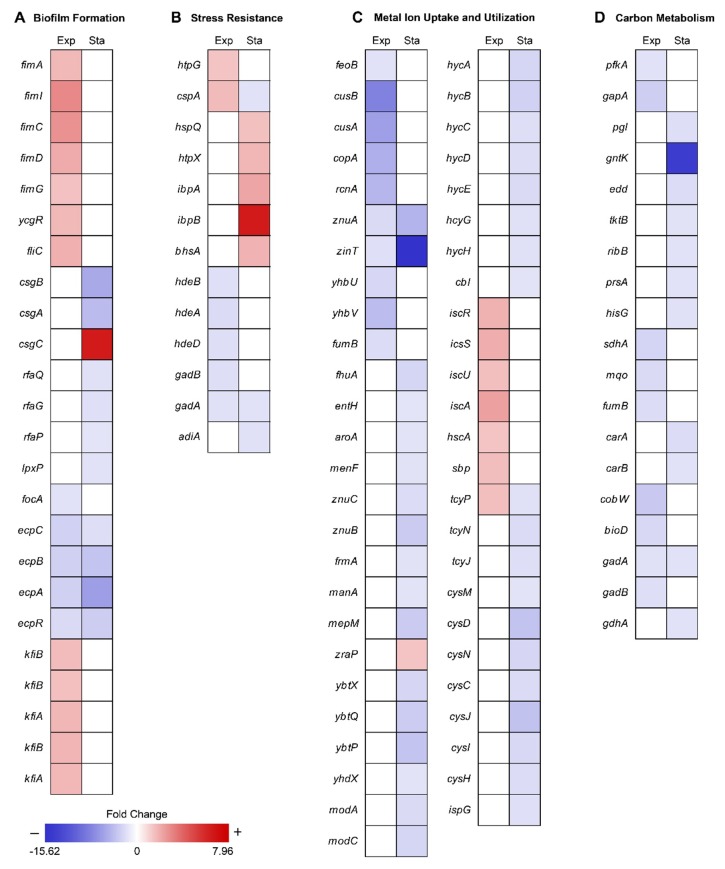
Hierarchical clusters diagram of the fold change ratio of DEGs for each group. Each column represents the DEGs in the exponential (Exp) and stationary (Sta) growth phase, respectively. The fold change is relative to the original value. Mean positive fold change values represent the expression ratio of MG to NG for the DEGs up-regulated under MG conditions compared to that under NG conditions and are indicated in red. Mean negative fold change values represent the expression ratio of NG to MG for the DEGs down-regulated under MG conditions compared to that under NG conditions and are indicated in blue. The gene *csgC* was expressed only under MG conditions during the stationary growth phase.

To reveal the metabolic changes of EcN in response to decreased gravity, we performed comparative transcriptomic profiling (RNA-seq) during exponential and stationary growth phases under MG and NG conditions. Differentially expressed genes (DEGs) were selected using the criteria of ≥2-fold changes (P ≤ 0.01) in fragments per kilobase of exon per million fragments mapped (FPKM) values. As many genes in the EcN reference strain CP007799.1 from the National Center for Biotechnology Information (NCBI) database were unannotated, we identified these unlabeled genes using Basic Local Alignment Search Tool (BLAST) software (blastp) [20] and publicly available web-based databases, such as the EcoCyc *E. coli* database (http://ecocyc.org/) [21] and Kyoto Encyclopedia of Genes and Genomes (KEGG) [22]. A total of 86 genes were differentially expressed during the exponential growth phase and 224 genes were differentially expressed during the stationary growth phase (Figure 1C). Among these DEGs, 11 were common to both growth phases (Figure 1C). As shown in Table 1, the majority of common DEGs maintained their tendencies of being either up-regulated or down-regulated in both phases. Interestingly, some genes, such as cytochrome *bo*_3_ ubiquinol oxidase (*cyoA*), cold shock protein (*cspA*), and cysteine transporter (*tcyP*), exhibited changes in their direction of regulation. Among the DEGs in the exponential and stationary growth phases, 38 and 43 genes were up-regulated, respectively, while 48 and 181 genes were down-regulated, respectively (Tables S1 and S2). Some were represented according to functional categories of hierarchical clusters diagrams based on expression ratios of MG to NG (Figure 2). These categories included biofilm formation, stress responses, metal ion transport/utilization, and carbon metabolism.

### 2.2. Effect of MG on EcN Biofilm Formation

Cell surface components, such as type I curli fimbriae and lipopolysaccharide (LPS), are important for the formation of complex biofilm structures, which protect cells from exterior factors [23,24,25]. Previous research has shown that MG can induce changes in gene expression related to biofilm formation [4,9,12,26,27,28,29,30]. Similar to previous studies, most EcN genes related to type I fimbriae synthesis (*fimA, fimI, fimC, fimD, fimG*), flagella motility regulation (*ycgR*), and flagella synthesis (*fliC*) were up-regulated (2.18–4.14-fold) under MG during the exponential growth, which allowed for increased biofilm formation (Figure 2A and Table S1). However, these genes were not differentially expressed between conditions of MG and NG during stationary growth. Since oxygen availability is lower in MG than NG [31], cells may offset this difference by forming thicker and more structured biofilm architecture from the onset, regardless of growth phase [28]. Unlike other bacteria, EcN under MG during stationary growth phase repressed genes related to major structural subunit of curli (*csgB, csgA;* 5.19–6.55-fold), LPS core synthesis (*rfaQ, rfaG, rfaP*; 2.03–2.39-fold), and lipid A (*lpxP*; 2.25-fold) [32]. *CsgC*, which functions as an inhibitor of *csgA* expression was highly expressed under MG conditions (Figure 2A and Table S2) [33].

In addition to the structural robustness of the cell itself, the adhesion ability of EcN appeared to be the opposite of that reported for other gut microbiome species. For instance, F1C fimbriae, *E. coli* common pili (ECP), and K5 capsule play key roles in the adhesion of EcN to gut epithelial cells in [34]. Under MG, *focA*, which encodes the major F1C fimbriae subunit, was repressed during the exponential growth phase (2.28-fold) and *ecpC, ecpB, ecpA,* which encode ECP, and *ecpR*, which encodes the transcriptional regulator of ECP synthesis, were repressed (2.48–7.36-fold) during entire cell growth. Additionally, multiple copies of *kfiA* and *kfiB* encoding K5 capsule biosynthesis components were up-regulated (2.25–2.61-fold) under the same conditions (Tables S1 and S2). When *kfiB* is knocked-out, EcN attachment to Caco-2 cells is known to increase [35]. Collectively, the shifts in expression we observed may interfere with EcN adhesion to epithelial cells and prevent intestinal colonization. Additional experimental validation may need to be performed outside Earth in the future. Considering that cell adhesion of pathogens is increased under MG conditions [2], these results suggest that EcN may be less competitive in MG, resulting in pathogens causing an imbalance of the gut microbiome during spaceflight [34].

### 2.3. Effect of MG on EcN Stress Responses

**Figure 3:**
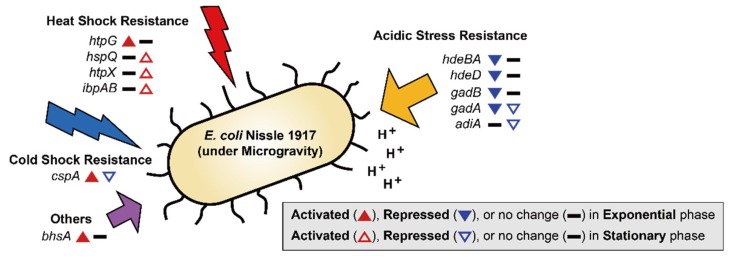
DEGs related to stress resistance are shown. The up-regulated and down-regulated genes under the MG condition compared to the NG condition are depicted by the direction of the up and down arrowheads, respectively. DEGs during the exponential and stationary growth phases are indicated by the solid and empty arrowheads, respectively.

Exposure to MG can result in several changes outside of cell membrane, including convectional thermal loss and reductions in shear force. Cells must adapt to new conditions under MG by expressing resistance-related proteins [36]. In previous studies, bacteria in MG showed increased resistance to heat, osmotic pressure, and acidic conditions [6,8,9,12,37]. EcN for the most part maintained these general stress response tendencies (Figure 2B and Figure 3). During the exponential growth phase, EcN in MG displayed higher expression levels of heat shock protein (*htpG*; 2.06-fold), cold shock protein (*cspA*; 2.38-fold) compared to those under NG conditions (Table S1). Expression of heat shock proteins (*hspQ*, *htpX*, and *ibpAB*) and membrane protein involved in stress response (*bhsA*) were increased (2.21–7.96-fold) in the stationary growth phase. Meanwhile, *cspA*, which was induced during exponential growth, was down-regulated (2.24-fold) during the stationary growth phase (Table S2). While *cspA* has a role as a global regulator [38], it is unclear why its expression tendency differed under the two growth phases.

Interestingly, EcN exhibited different trends in acidic resistance during the exponential growth phase under MG compared to that of other bacteria, with the repression of acid resistant chaperones (*hdeBA* and *hdeD*) being 2.38–2.75-fold [39] and glutamate decarboxylases (*gadB* and *gadA*) being 2.36–2.48-fold (Table S1). *Gad* encoded proteins are known as the components of the glutamate-dependent acid resistance (AR) system, which is an efficient AR system called AR2 [40]. During the stationary growth phase, glutamate decarboxylase (*gadA*) remained repressed (2.27-fold), along with arginine decarboxylase (*adiA*; 2.30-fold), which is another AR system known as AR3 (Table S2) [41]. These findings suggest that MG conditions may retard the growth of EcN by inducing more acidic conditions compared to that of NG, especially during the exponential growth phase [42]. Lower resistance to acidic stress by EcN under conditions of MG indicates that EcN may not function efficiently as a probiotic during spaceflight when administered orally, as it must pass through the gastrointestinal tract.

### 2.4. Effect of MG on EcN Metal Ion Transport/Utilization

**Figure 4:**
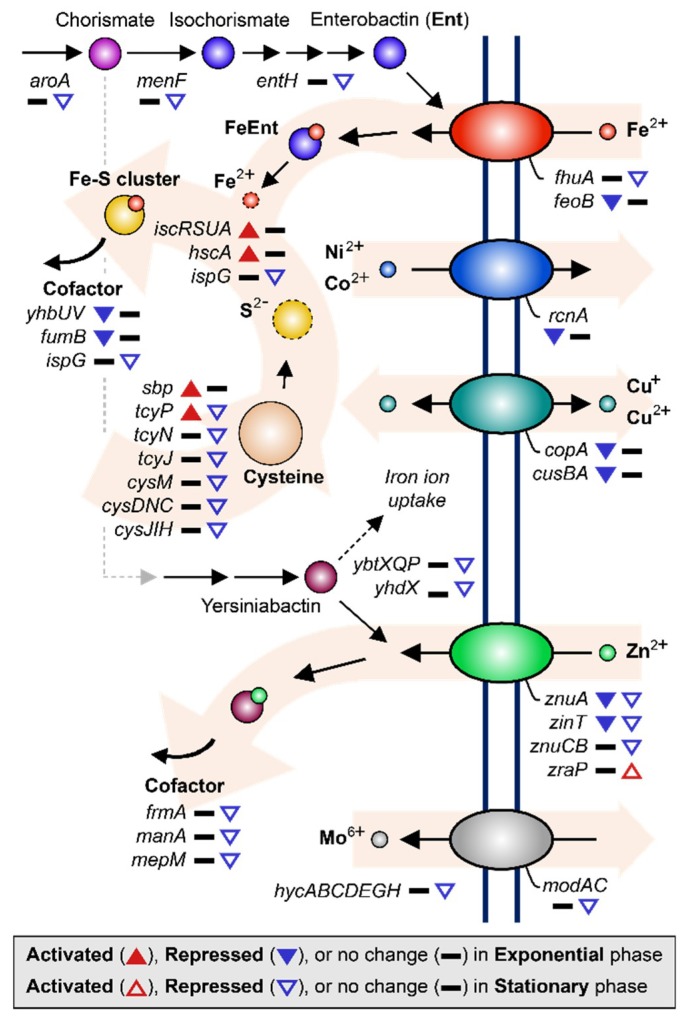
DEGs related to metal ion acquisition and utilization are shown. The up-regulated and down-regulated genes under the MG condition compared to the NG condition are depicted by the direction of the up and down arrowheads, respectively. DEGs during the exponential and stationary growth phases are indicated by the solid and empty arrowheads, respectively.

Previous studies have commonly shown that MG induces repression of metal ion uptake systems, including those for iron [9], copper [8], nickel [7], and zinc [9]. Similar to these previous studies, we found that various metal ion uptake systems in EcN were also down-regulated under MG conditions (Figure 4). During the exponential growth phase, ferrous ion transporter (*feoB*) was repressed 2.30-fold, the copper ion efflux system (*cusBA*) 7.32–9.58-fold, copper transport chaperone (*copA*) [43] 6.07-fold, nickel efflux protein (*rcnA*) 5.53-fold, zinc transporter (*znuA*) 2.82-fold, and Zn/Cd binding protein (*zinT*) 2.43-fold. Expression of enzymes that require iron as a cofactor, such as ubiquinone biosynthesis protein (*yhbUV*) and succinate dehydrogenase (*fumB*), was also reduced 2.66–5.10-fold as a result of less iron uptake (Figure 2C and Table S1) [44].

During stationary growth, the tendencies of metal ion uptake regulation continued. Genes related to ferrichrome transporter protein synthesis (*fhuA*) and enterobactin biosynthesis (*entH*, *aroA*, and *menF*) were down-regulated 2.05–3.20-fold. Enterobactin is a siderophore that transports iron ions by binding the ion and then passing through the cellular membrane together [45,46]. Similarly, synthesis of zinc transporter (*znuA* and *znuCB*) and Zn/Cd binding protein (*zinT*) were continuously down-regulated 2.68–15.62-fold along with other enzymes that require zinc ions as cofactors (*frmA*, *manA*, and *mepM*), which were down-regulated 2.12–3.96-fold [47,48,49]. Zinc uptake modulator (*zraP*) blocks zinc ion uptake and was up-regulated 2.11-fold [50]. It is worth pointing out that the synthesis of yersiniabactin transporters (*ybtX*, *ybtQ*, *ybtP*, and *yhdX*), which are known as the siderophores transporting iron and zinc ions in the EcN [51,52], were repressed 2.09–4.50-fold during the stationary growth phase. In addition, the molybdate efflux system, including molybdate ion transporter (*modA* and *modC*) and *hycA, hycB, hycC, hycD, hycE, hycG,* and *hycH*, which are known to be dependent on molybdate [53,54], were expressed less under MG during the stationary growth phase (2.32–3.48-fold) compared to that under NG (Figure 2C and Table S2).

The tendency to lower free metal ion concentrations in the cytoplasm of cells was observed according to the utilization of iron ions. Intracellular iron ions may remain as free cations or form complexes, such as iron–sulfur (Fe-S) clusters or heme groups [55,56]. By forming Fe-S complexes, imported free iron ions associate with sulfate ions to alleviate the exposure of cells to hydroxide radicals that result from the presence of excessive free iron ions [57]. During the exponential growth phase, genes related to Fe-S assembly, such as iron–sulfur cluster assembly protein synthesis (*iscR, iscS, iscUA*) and iron–sulfur cluster biosynthesis chaperone (*hscA*), were up-regulated 2.05–3.31-fold. To supply sulfur for combining with iron ions, cysteine can be broken down by cysteine desulfurase [58,59]. Therefore, elevated assembly of the Fe-S clusters may induce sulfur and cysteine starvation inside the cell, resulting in the activation of sulfate transporter (*sbp*), which was up-regulated 2.32-fold, and cysteine transporter (*tcyP*), which was up-regulated 2.27-fold (Figure 2C and Table S2) [59,60].

However, during the stationary growth phase, the direction of regulation of genes related to iron utilization was the opposite of that during exponential growth. For instance, sulfur metabolism was generally down-regulated. As shown in Figure 2C and Table S2, repressed genes included transcription factor *cbl* (2.07-fold), cysteine transporter (*tcyP*, *tcyN*, *tcyJ*; 2.30–2.69-fold), cysteine synthase (*cysM*; 2.13-fold), and genes related to sulfate assimilation (*cysDNC* and *cysJIH*; 2.61–4.63-fold) [61]. These genes are known to be activated by transcription factor *cysB* when induced by O-acetylserine (OAS) [59]. Furthermore, sulfide and thiosulfate act in a competitive manner with OAS as anti-inducers [62]. These results indicate that the intracellular availability of sulfide and thiosulfate during stationary growth of the EcN under MG conditions may be the opposite to that during exponential growth.

### 2.5. Effect of MG on EcN Carbon Metabolism

**Figure 5:**
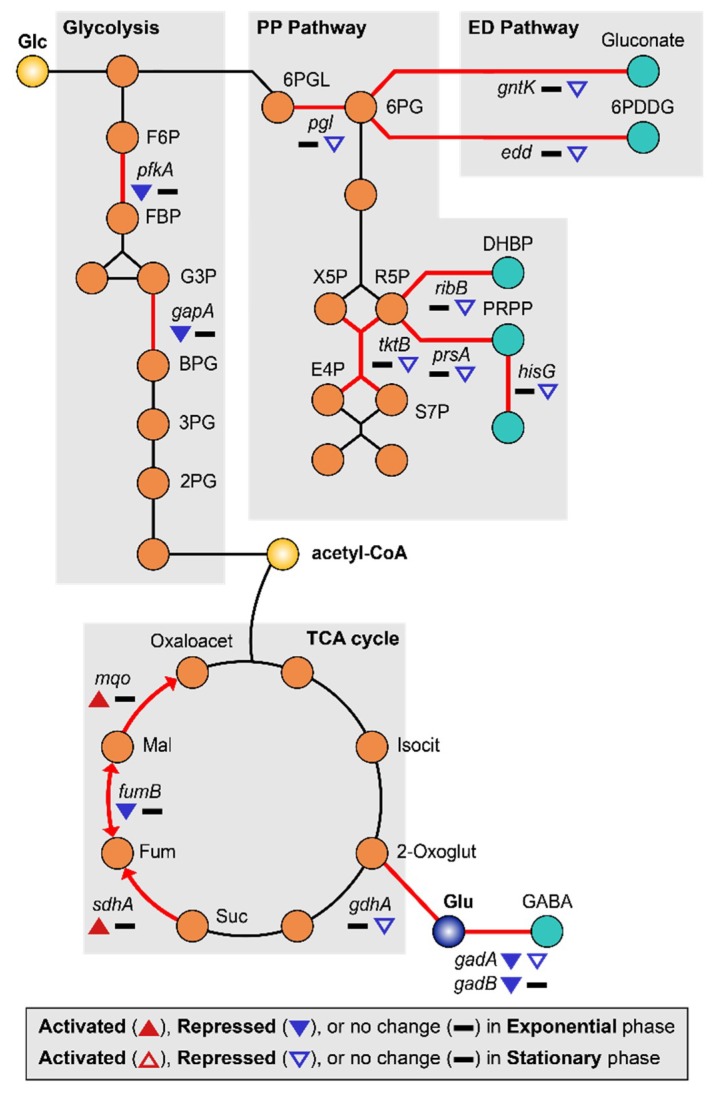
DEGs related to carbon starvation metabolism are shown. The up-regulated and down-regulated genes under the MG condition compared to the NG condition are depicted by the direction of the up and down arrowheads, respectively. DEGs during the exponential and the stationary growth phases are indicated by the solid and empty arrowheads, respectively.

MG is able to induce a lack of convective mixing, resulting in the creation of what have been referred to as “depletion zones” [63]. Many studies have reported that MG induces carbon starvation stress due to nutrient depletion [7,9,26]. Under conditions of carbon starvation, carbohydrate catabolism was altered, especially the sequentially inhibition of glycolysis and the pentose phosphate (PP) pathway [64]. Consistent with this, we found that the glycolysis enzymes 6-phosphofructokinase (*pfkA*) and glyceraldehyde-3-phosphate dehydrogenase (*gapA*) were repressed (2.27–3.68-fold) during the exponential growth phase, while genes related to the pentose phosphate (PP) pathway (*pgl*, *tktB*, *ribB*, *prsA*, and *hisG*) and Entner–Doudoroff (ED) pathway (*gntK* and *edd*) [65] were repressed (2.17–14.89-fold) during the stationary growth phase (Figure 5, Tables S1 and S2). Repression of *pfkA* at the exponential growth phase can induce the higher flux of glucose to the PP or ED pathway. Activation of these pathways may help protect cells from oxidative stress by production of NADPH [66,67]. By the way, regulation of the tricarboxylic acid (TCA) cycle can burden oxidative stress to the cell. Repressed glycolytic metabolism due to carbon starvation may also affect the tricarboxylic acid (TCA) cycle [68]. During the exponential growth phase, succinate:quinone oxidoreductase (*sdhA*) and malate:quinone oxidoreductase (*mqo*) were up-regulated 2.76–3.24-fold, concurrently with the repression of fumarate hydratase (*fumB*) 2.68-fold and fumarate reductases 2.77–3.03-fold (Figure 5, Tables S1 and S2). The reduction of quinone by increased expression of *mqo* may be especially detrimental to EcN as the imbalance between quinone and quinol can induce oxidative stress [69]. In addition, there is a possibility of fumarate accumulation due to the activation of *sdhA*, which may consequently result in oxidative stress [70,71].

Other genes related to energy metabolism were also differentially regulated. During the exponential phase, vitamin synthesis (*cobW*) and biotin synthesis (*bioD*) genes were down-regulated 2.96–4.17-fold (Table S1). Since biotin is an essential cofactor for fatty acid metabolism [72], a shortage of biotin under MG may hamper cell growth. During the stationary growth phase, pyrimidine synthesis enzymes (*carA* and *carB*) were repressed 2.27–2.65-fold. Furthermore, the γ-aminobutyric acid (GABA) shunt pathway was also repressed, along with the acid resistance system (Figure 5) [73,74]. Finally, *gadA* and *gadB* were repressed 2.36–2.48-fold during the exponential growth phase while *gadA* and *gdhA* were repressed 2.27–2.28-fold during the stationary growth phase (Figure 2D, Tables S1 and S2).

## 3. Conclusions

Probiotics, including EcN, may be an effective solution in the management of the gut microbiome of astronauts as they can protect the gut microbiome from pathogens during the space flight. We investigated the effects of simulated MG on EcN using comparative transcriptomic analysis during exponential and stationary growth phases. Some changes identified were disadvantageous to EcN when competing against other pathogenic microbes. EcN tended to develop lower relative abilities for acidic resistance and adhesion under MG conditions, which may make it more difficult for it to survive as it passes through the acidic conditions of stomach and to attach to intestinal cells. Additionally, EcN exhibited altered metal ion transport/utilization and repressed energy and carbon metabolism in response to oxidative stress and nutrient depletion, respectively. Collectively, these results demonstrate the need for further studies that will be essential for the development of effective probiotics that are able to function under MG as guardians of the gut microbiome.

## 4. Materials and Methods

### 4.1. Overall Work Flow of This Study

The total work flow of our study was listed in the following order. (1) Culture the sample under microgravity and normal gravity conditions, respectively. (2) Harvest both samples at the exponential and stationary growth phase. (3) Extract total RNAs from the harvested samples. (4) Remove the rRNA from the extracted RNA samples. (5) Construct the RNA-seq library samples. (6) Run next-generation sequencing and analyze the sequencing data.

### 4.2. Bacterial Strain and Growth Conditions

The strain used in this study was EcN, which was cultured in M9 minimal media (47.8 mM Na_2_HPO_4_∙7H_2_O, 22 mM KH_2_PO_4_, 8.6 mM NaCl, 18.7 mM NH_4_Cl, 2 mM MgSO_4_, and 0.1 mM CaCl_2_) supplemented with 0.4% glucose (*w*/*v*). To develop the inoculum, a single colony of EcN grown on a Luria-Broth (LB) broth agar plate was inoculated into M9 media with 0.4% glucose and incubated with shaking at 37 °C. The cultures were incubated overnight to allow the cells to enter the stationary growth phase and then used as inocula for the clinostat system.

### 4.3. Growth of EcN in a Clinostat

After culturing overnight, and the EcN cells entering the stationary growth phase, a sample of the culture was diluted 10^−3^ and added to clinostat vessels (Synthecon, Inc., Houston, TX, USA). The vessels (d = 12.5 cm) were installed either perpendicularly or horizontally to simulate MG and NG conditions, respectively. To generate conditions of low shear stress, samples were incubated in vessels rotating < 25 rpm. The optical density at 600 nm (OD_600_) was checked to monitor the growth profile of EcN for each condition. Briefly, the clinostat vessel was detached from the instrument every hour and the OD_600_ of the culture was determined to generate the growth profiles of the EcN. Based on the growth curves, we determined the points of exponential and stationary growth. For RNA extraction, 1 mL of EcN was harvested at two points of cell growth, which included the exponential growth phase (OD_600_ 0.6–0.8) and stationary growth phase (OD_600_ 1.2). Each experimental condition was performed in duplicate.

### 4.4. RNA Extraction, cDNA Synthesis, and Sequencing

Total RNA was extracted from 3 mL of cultured EcN samples at each growth point for each gravitational condition. Before RNA extraction, harvested samples were treated with RNAprotect Bacteria Reagent (Qiagen, Hilden, Germany). Then, we used Qiagen RNeasy Plus Mini kit (Qiagen, Hilden, Germany) for RNA extractions. First, 6 mL of RNA protect reagent was added to each culture sample. After 5 min incubation, the samples were pelleted and resuspended in 400 μL Tris-EDTA pH 8.0 (TE) buffer (Sigma–Aldrich, Saint Louis, MO, USA). After a second round of pelleting and washing, the sample was eluted with 200 μL TE buffer. Then 1 μL SUPERase•In RNase inhibitor (Invitrogen, Carlsbad, CA, USA), 3 μL of 10 M units of Ready-Lyse Lysozyme solution (Lucigen, Middleton, WI, USA), 1 μL of 20 mg/mL Proteinase K (Invitrogen, Thermo Fisher Scientific Inc, Waltham, MA, USA), and 3 μL of 20% sodium dodecyl sulfate (Sigma–Aldrich, Saint Louis, MO, USA) were added and incubated for 30 min in ice. Then, 700 μL RLT-β-ME buffer, which was prepared with addition of 10 μL of β-mercaptoethanol (Sigma–Aldrich, Saint Louis, MO, USA) to 1 mL of RLT Buffer, was added to make visible particle. The samples were centrifuged and the supernatants purified using gDNA Eliminator Mini Spin Columns (Qiagen, Hilden, Germany). The filtered samples then had 500 μL of 100% EtOH added and purified using RNeasy Mini Spin Columns (Qiagen, Hilden, Germany). Using 350 μL RW1 and 1 mL RPE buffer (Qiagen, Hilden, Germany), the RNA samples were washed and eluted with RNase-free deionized distilled water. Purified RNA samples were analyzed using an RNA 6000 Pico Kit on an Agilent 2100 bioanalyzer (Agilent, Santa Clara, CA, USA). The RNA integrity number (RIN) ranged from 2.6 to 9.9.

To remove any ribosomal RNA (rRNA) from the RNA stock, a Ribo-Zero rRNA Removal Kit for Gram-negative bacteria (Illumina, San Diego, CA, USA) was used. The RNA solutions were treated with 2 μL Ribo-Zero Reaction Buffer (Illumina, San Diego, CA, USA) and 5 μL Ribo-Zero Removal Solution (Illumina, San Diego, CA, USA). Magnetic beads were prepared by washing and then resuspending them in 30 μL AMPure XP magnetic bead resuspension solution (Beckman Coulter, Fullerton, CA, USA) and 3 μL of RiboGuard RNase Inhibitor (Illumina, San Diego, CA, USA). The prepared magnetic beads were then added to hybridized the RNA sample and purified using RNeasy MinElute spin columns (Qiagen, Hilden, Germany). Removal of the rRNA was confirmed using a Qubit Fluorometer (Invitrogen, Thermo Fisher Scientific Inc, Waltham, MA, USA) and Agilent 2100 Bioanalyzer (Agilent, Santa Clara, CA, USA).

The extracted RNA samples with the rRNA removed were then used for cDNA synthesis using a KAPA Stranded RNA-Seq Library Preparation Kit (Kapa Biosystems, Inc., Wilmington, MA, USA). Samples were fragmented and each strand synthesized into cDNA. After bead-based purification, the cDNA strands were A-tailed for adapter ligation. After the adaptor ligation incubation, the samples were purified using 20% PEG 8000/2.5 M NaCl solution (Kapa Biosystems, Inc., Wilmington, MA, USA). The cDNA strands of sizes consistent with the target range were selected and then amplified by real-time PCR. Overall, eight samples including duplicate samples from the two growth phases and two culture condition were quality-checked (9.86–64.4 ng/μL) using the Agilent 2100 Bioanalyzer (Agilent, Santa Clara, CA, USA) and sequenced using Illumina HiSeq 4000 System (Macrogen, Seoul, Korea). All raw data for RNA-seq has been deposited into the Gene Expression Omnibus at the NCBI under GSE147272.

### 4.5. Read Processing, Alignment, and Quantification

Sequenced reads from the RNA-seq analysis showed low proportions of rRNA, which indicated that the rRNA was successfully removed. The RNA-seq reads were then mapped based on the EcN reference genome (CP007799) using bowtie and cuffdiff tools. The calculated FPKM values from cuffdiff were used to identify the DEGs. DEGs with log_2_-fold expression changes ≥ 1.0 and false discovery rates (FDR) ≥ 0.01 were selected.

### 4.6. Differential Expression and Functional Analyses

Unidentified DEGs from the reference genome were compared with genes from other *E. coli* strains using blastp BLAST software and the EcoCyc database (www.ecocyc.org). The function of each DEG was then analyzed based on the KEGG and EcoCyc gene operon online databases.

## Figures and Tables

**Table 1:** DEGs presented in both MG and NG. All DEGs that overlapped the two gravity conditions are listed as fold change values and the categorized group indicated. Fold change value are relative to the original value. The positive fold change values are provided as the expression ratio of MG to NG. The negative fold change values are provided as the expression ratio of NG to MG.

Locus Tag	Fold Change (Exp)	Fold Change (Sta)	Gene Function	Gene	Function Category
ECOLIN_01835	−3.51	−2.48	hypothetical protein	*ecpC*	Biofilm formation
ECOLIN_01840	−3.57	−4.46	hypothetical protein	*ecpB*	Biofilm formation
ECOLIN_01845	−3.56	−7.36	fimbrial protein	*ecpA*	Biofilm formation
ECOLIN_01850	−2.76	−3.78	LuxR family transcriptional regulator	*ecpR*	Biofilm formation
ECOLIN_19660	2.38	−2.24	cold-shock protein	*cspA*	Stress resistance
ECOLIN_19450	−2.36	−2.27	glutamate decarboxylase	*gadA*	Stress resistance
ECOLIN_09330	2.27	−2.30	L-cystine transporter tcyP	*tcyP*	Metal ion utilization
ECOLIN_09975	−2.82	−5.68	zinc ABC transporter substrate-binding protein	*znuA*	Metal ion utilization
ECOLIN_10835	−2.43	−15.62	zinc/cadmium-binding protein	*zinT*	Metal ion utilization
ECOLIN_02515	2.50	−2.17	cytochrome o ubiquinol oxidase subunit II	*cyoA*	Uncategorized
ECOLIN_01860	−3.45	−11.11	50 S ribosomal protein L31 type B	*-*	Uncategorized

## Supplementary Materials

Supplementary Materials can be found at https://www.mdpi.com/1422-0067/21/8/2666/s1. Table S1: Differentially Expressed Genes of the *Escherichia coli* Nissle 1917 strain in the exponential growth phase, Table S2: Differentially Expressed Genes of the *Escherichia coli* Nissle 1917 strain in the stationary growth phase.
